# NICUs in the US: levels of acuity, number of beds, and relationships to population factors

**DOI:** 10.1038/s41372-023-01693-6

**Published:** 2023-05-19

**Authors:** Roberta Pineda, Kati Knudsen, Courtney C. Breault, Elizabeth E. Rogers, Wendy J. Mack, Alicia Fernandez-Fernandez

**Affiliations:** 1 Chan Division of Occupational Science and Occupational Therapy, University of Southern California, Los Angeles, CA USA; 2 Keck School of Medicine, Department of Pediatrics, Los Angeles, CA USA; 3 Gehr Family Center for Health Systems Science and Innovation, University of Southern California, Los Angeles, CA USA; 4 Program in Occupational Therapy, Washington University, St. Louis, MO USA; 5 Neonatal Intensive Care Unit, Providence St. Vincent Medical Center, Portland, OR USA; 6 California Perinatal Quality Care Collaborative (CPQCC), Division of Neonatal and Developmental Medicine, Department of Pediatrics, Stanford University School of Medicine, Stanford, CA USA; 7 Department of Pediatrics, University of California San Francisco, San Francisco, CA USA; 8 Department of Population and Public Health Sciences, University of Southern California, Los Angeles, CA USA; 9 Physical Therapy Department, Dr. Pallavi Patel College of Health Care Sciences, Nova Southeastern University, Fort Lauderdale, FL USA; 10 Neonatal Intensive Care Unit, South Miami Hospital, Miami, FL USA

**Keywords:** Rehabilitation, Medical ethics, Health care economics

## Abstract

**Objective:**

To 1) define the number and characteristics of NICUs in the United States (US) and 2) identify hospital and population characteristics related to US NICUs.

**Study design:**

Cohort study of US NICUs.

**Results:**

There were 1424 NICUs identified in the US. Higher number of NICU beds was positively associated with higher NICU level (*p* < 0.0001). Higher acuity level and number of NICU beds related to being in a children’s hospital (*p* < 0.0001;*p* < 0.0001), part of an academic center (*p* = 0.006;*p* = 0.001), and in a state with Certificate of Need legislation (*p* = 0.023;*p* = 0.046). Higher acuity level related to higher population density (*p* < 0.0001), and higher number of beds related to increasing proportions of minorities in the population up until 50% minorities. There was also significant variation in NICU level by region.

**Conclusions:**

This study contributes new knowledge by describing an updated registry of NICUs in the US in 2021 that can be used for comparisons and benchmarking.

## Introduction

There were just over 3.6 million live births in the United States (US) in 2020, and although pinpointing the number of neonatal intensive care unit (NICU) admissions is a challenge, it has been estimated that 9–13% of infants require neonatal intensive care for complex medical needs [1–3]. However, these estimates are likely low, as birth certificates may not be a completely reliable source of NICU admission data [4, 5]. Additionally, NICU admission rates are anticipated to grow due to preterm births and maternal medical conditions such as gestational diabetes [6]. However, the number of NICU beds in a community is important, as it is related to increased NICU utilization [7]. Despite the critical role of NICUs in the US health care system, there is no current, updated, and comprehensive catalog or registry containing all US NICUs.

There are many different types of NICUs (level, size, type of associated hospital) across the US. States have individual protocols for certifying bed space that vary in criteria [8]. Certificate-of-need (CON) legislation can limit the addition of hospital beds, based on other resources/available beds in the immediate community [8, 9]. Although some states have CON legislation, CON applies to all health care service delivery in the state, and it does not always, or in some instances ever, focus specifically on pediatrics or NICUs. CON legislation aims to lower healthcare costs by limiting duplicative medical services and requiring hospitals to receive approval before expanding the number of beds available [9, 10]. One study found that states without CON legislation had more hospitals with NICUs and a higher total number of beds, as well as increased infant mortality rates [9]. Another study found that a higher number of NICU beds does not necessarily equate to improved or equitable access to care, as higher numbers of NICU beds were not necessarily located in areas with higher rates of low birthweight infants [11].

NICUs also vary in acuity level. Prior to 2012, the level of NICUs had been categorized as level II or III in the US [12]. This was expanded to the current classification that labels NICUs as level II, III, or IV depending on the capacity to care for infants with different degrees of medical and surgical complexity. There are no federal policies defining medical services provided within levels of NICUs, and each state is able to set their own guidelines about NICU levels of care [8, 13]. NICUs can apply and be surveyed to verify their level through the American Academy of Pediatrics (AAP) [14]. In 2012, the AAP released a policy statement outlining proposed levels of care [15], but states have adopted variations of this policy [13].

Research has demonstrated that the NICU where an infant receives care is important, especially for high-risk infants with complex medical conditions [16, 17]. Further, NICU level and patient volume affect infant outcomes [18, 19]. While lower-risk cases may be appropriate to receive care at a Level II, or community-based NICU, there is also data that has shown higher risk of developing serious conditions such as bronchopulmonary dysplasia if hospitalized at a Level II or III NICU compared to a Level IV NICU [20]. Despite this, there has been a proliferation of NICUs, and the shifting of higher-risk cases from the high-volume level III or IV NICUs to smaller community NICUs [12]. Better understanding the scope of NICUs across different areas of the US can aid in our understanding of NICU proliferation and lead to positive changes to improve outcomes for high-risk infants and families in the future.

Population-based features of the area in which a NICU is located may also be related to different NICU characteristics. It could be assumed that higher population density will necessitate more need for NICU beds in a given area. The risk of preterm birth is highest among Black families [21] and among those of lower socioeconomic status [22]. However, despite the likelihood of higher need for NICU beds in areas with high proportions of racial minorities and those living in poverty, there are also studies that have demonstrated health inequities, with fewer available medical facilities for families from diverse or poor backgrounds [23]. It is not well understood what population factors are related to more or less access to NICU care.

The objectives of this study were to 1) define the number of NICUs in the United States (US) and their size, acuity level and demographic characteristics and 2) identify hospital and population characteristics related to US NICUs.

## Methods

The institutional review board of the University of Southern California formally determined this study did not meet criteria for human subjects research.

This was a study employing several forms of data collection which included two neonatal therapy surveys, NICU databases, websites, phone calls, and online correspondence with hospital staff. Population databases were also used to extract information about the location (city or county) in which each NICU was located.

### Neonatal therapy surveys

Two neonatal therapy surveys were conducted in 2016 and 2020, and respondents identified their hospital characteristics (hospital name, location, level, and number of beds) [24, 25]. This information was extracted to establish each hospital as its own ‘participant’, representing each US-based NICU. The initial database identifying hospitals in which neonatal therapy staffing data was available was expanded to identify exhaustive information about each NICU in the US.

### NICU databases

Following the two surveys, a list of all the represented hospitals was constructed. This was then checked against other lists of NICUs in the United States, including a list published by the AAP [26], as well as a database publicly available by Neonatology Solutions [27]. These were then cross-checked with available information from the Vermont Oxford Network [28] and the Children’s Hospitals Consortium [29]. Attempts were made to integrate lists of NICUs from the American Hospital Association (AHA);[30] however, while helpful in identifying which hospitals had NICUs, the number of beds were at least sometimes inflated due to having other levels of care or multi-use beds included in the total count, potentially largely in Level II or small rural NICUs. Additional lists from AHA became available during the latter part of this study, but they were restricted unless payment was made to access them which was not possible for this unfunded work. Data also was limited to NICU, number of beds, and number of ventilators. Although 1367 NICUs were reported in a previous manuscript [25], since this publication an additional list was incorporated in the final number reported in this manuscript [31]. The additional lists used came from cross-checking with the National Network of Perinatal Quality Collaboratives list [31], which identified NICUs in each state. This added 60 NICUs to the list, with those additions largely being small, level II NICUs. It is important to note that these lists also included many hospitals which did not have NICUs, requiring direct phone call confirmation to remove such hospitals from the list.

Different information was available from each source and included the level of NICU (level II, III, or IV), type of NICU (academic medical center, community, regional non-academic, county, Indian health services, military hospital), whether part of a children’s hospital (freestanding children’s hospital, not part of a children’s hospital, children’s hospital within a hospital), type of organization (nonprofit, corporation/proprietary, governmental), and number of approved beds. The level of NICU was defined as the highest level of care available at that hospital, with the number of approved beds being the maximal number of beds that can be occupied by infants with complex medical needs at any level of care.

### Phone and online information

When information was missing or inconsistent, internet searches and phone calls to the NICU were completed by research assistants. Information was documented from hospital web sites and from internet searches. During phone calls, information was asked of the person answering the call, and return calls were requested as appropriate based upon the responses from each site. Most often when information was unknown by the office assistants answering the phone, the call was directed to charge nurses or NICU or hospital leadership who provided the information. When contact information was given to the research team from phone calls, online correspondence was used through messaging and email to gain hospital-specific information. Information from healthcare professionals at specific hospitals was also sought in online communities. Phone calls and online correspondence also aided in defining NICUs that had closed. When there were inconsistencies across different sources of information (survey, database), verification from an employee at each NICU was sought.

### Population and demographic factors

The NICU surveys, phone calls, and databases identified the location (state and city) of each US-based NICU. Location was further categorized by region (Northeast, Southeast, Midwest, Southwest, and West) for each city in which a NICU was located (based on natural city boundaries, not including inner ring suburbs). Population density (the number of people per square mile) and the proportion of the population living below the poverty line (currently $26,246 for a family of 4) were collected from World Population Review 2019 projections (and 2015 projections, when 2019 projections were unavailable) [32]. Also for each city in which a NICU was located, the percentage of the population under the age of 5 years old (a measure to determine the need for specialized pediatric medical services in an area) and the percentage of the population being from ethnic and racial minorities (Black, American Indian or Alaskan, Asian, Native Hawaiian or Pacific Islander, Hispanic or Latino, or 2 or more races) were defined from US Census Bureau American Community Survey, 2015–2019 data [33]. When information for the city was not available, the county’s information was used.

Further, we defined whether each NICU was located in a state subject to CON legislation using previously published work [9].

A comprehensive spreadsheet was developed from the multiple sources of information previously mentioned. Discrepancies across sources were reconciled using hospital websites and phone calls to hospital staff. Duplicates were removed. A member of the research team then double-checked 10% of the data to ensure accuracy.

An additional spreadsheet was populated with NICU information for each identified city. Each city was put in context of total number of NICU beds in the city, number of NICUs at each level, and population factors (as above) related to each city.

### Statistical analysis

Descriptive statistics were used to define the number of NICUs in the US according to type, size, and level. Hospital and population factors related to NICU number of beds were investigated using linear regression models. The dependent variable of number of NICU beds was log-transformed for normality. Categorical independent variables (type of hospital, whether part of a free-standing children’s hospital, type of organization, region, subject to CON legislation) were modeled as factor variables, represented by indicator variables relative to a referent level. Continuous and ordinal variables (NICU level, population density, percentages of population living below poverty line, under 5 years old and or ethnic/racial minority) were graphically evaluated for linearity of the relationship with log(number of NICU beds) using lowess plots (a smoothing plot of the independent versus the dependent variable). Linear splines (which fits separate regression slopes at different levels of the independent variable) were used when non-linearity was present. Independent variables were evaluated for collinearity using correlations and variance inflation factor. Each independent variable was first analyzed separately for its association with log(number of NICU beds). An initial multivariable linear regression model included all independent variables that were univariately statistically significantly associated with log(number of NICU beds) at 2-sided *p* < 0.05. Independent variables were retained if they remained statistically significant in the multivariable model. Independent variables that were not significantly associated with log(number of NICU beds) on univariate modeling were added into the multivariable model to test for statistical significance. The final multivariable model included independent variables that retained statistical significance when adjusted for all other variables in the model. Model residuals met assumptions of normality. Linear regression models were presented as regression coefficients with 95% confidence intervals, p-values, and model R^2^. The same hospital and population independent variables were evaluated for their association with NICU level (II, III, IV) using ordinal logistic regression; the same modeling approach for selection of independent variables in the multivariable model was used. Unadjusted and multivariable ordinal regression results are presented as odds ratios with 95% confidence intervals and *p*-values; odds ratios greater than 1 indicate positive associations of an independent variable with higher NICU level, while odds ratios less than 1 indicate inverse associations of an independent variables with lower NICU level. Data were further reorganized to reflect city-specific NICU information including number of NICUs, total number of beds across those NICUs, in addition to region, population density, percentage of population less than 5 years, and the percentage of the population belonging to racial and ethnic minorities. The city-based analysis related population factors to log(number of beds) using the same linear regression methods summarized above. Analyses were conducted using Stata 17 (StataCorp, 2021. Stata Statistical Software: Release 17. College Station, TX: StataCorp LLC).

## Results

There were 1874 hospitals identified as having NICUs (through survey responses and the NICU databases). Four hundred fifty were confirmed to not have an operational NICU (41 had closed; 409 did not have a NICU, despite being on a NICU list). Therefore, 1424 currently operational NICUs in the US were identified, of which 570 (40%) were level II, 702 (49%) were level III, and 152 (11%) were level IV.

**Fig. 1:**
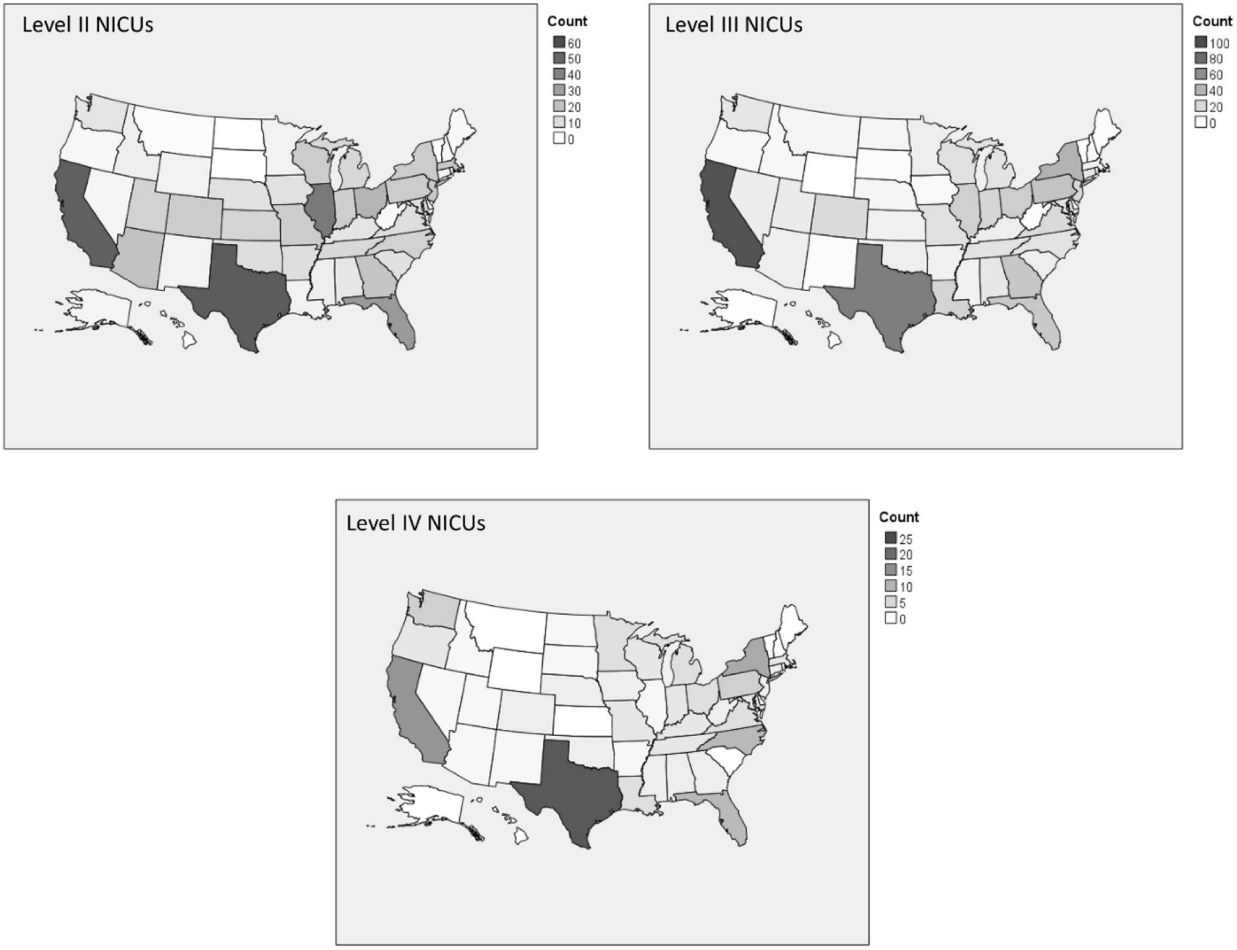
Number of different level NICUs in each state.

See Fig. 1 for the number of different level NICUs in each state. The numbers of NICUs are concentrated in large, high-density states such as California and Texas. There are also a higher number of NICUs in the Eastern and Midwestern portions of the US.

The NICUs ranged from 1 to 173 beds with a median of 18 (IQR 9.5–34) beds. Level II NICUs ranged from 1 to 40 beds with a median (IQR) of 8 (6–12) beds; level III NICUs ranged from 4 to 98 beds with a median (IQR) of 25 (16–37) beds; and level IV NICUs ranged from 15 to 173 beds with a median (IQR) of 55 (42–72) beds.

Table 1 lists characteristics of the US-based NICUs. There were more NICUs concentrated in the Southeast (making up 26% of NICUs) than in other regions of the US. Most NICUs were part of a community hospital (79% of NICUs), are not part of a children’s hospital (85% of NICUs) and are nonprofit (68% of NICUs).

**Table 1:** Characteristics of the NICUs.

(*n* = 1424)	*N* (%)
*NICU Level*	
Level II	570 (40%)
Level III	702 (49%)
Level IV	152 (11%)
*Region*	
Northeast	258 (18%)
Southeast	367 (26%)
Midwest	314 (22%)
Southwest	174 (12%)
West	311 (22%)
*Type of Hospital (n* *=* *1416)*	
Academic Medical	177 (12%)
Community	1112 (79%)
Regional (non-academic)	82 (6%)
County	30 (2%)
Indian health services	3 (0.2%)
Military	12 (0.8%)
*Label of Children’s Hospital*	
Not part of a children’s hospital	1206 (85%)
Free standing children’s hospital	68 (5%)
Children’s hospital within hospital	150 (10%)
*Type of Control*	
Nonprofit	968 (68%)
Corporation/proprietary	253 (18%)
Governmental	179 (13%)
Unknown	24 (1%)

*N* = 1424 unless otherwise specified (due to missing data).

There were a total of 35,601 NICU beds (5,592 Level II, 20,631 Level III, and 9378 Level IV).

**Fig. 2:**
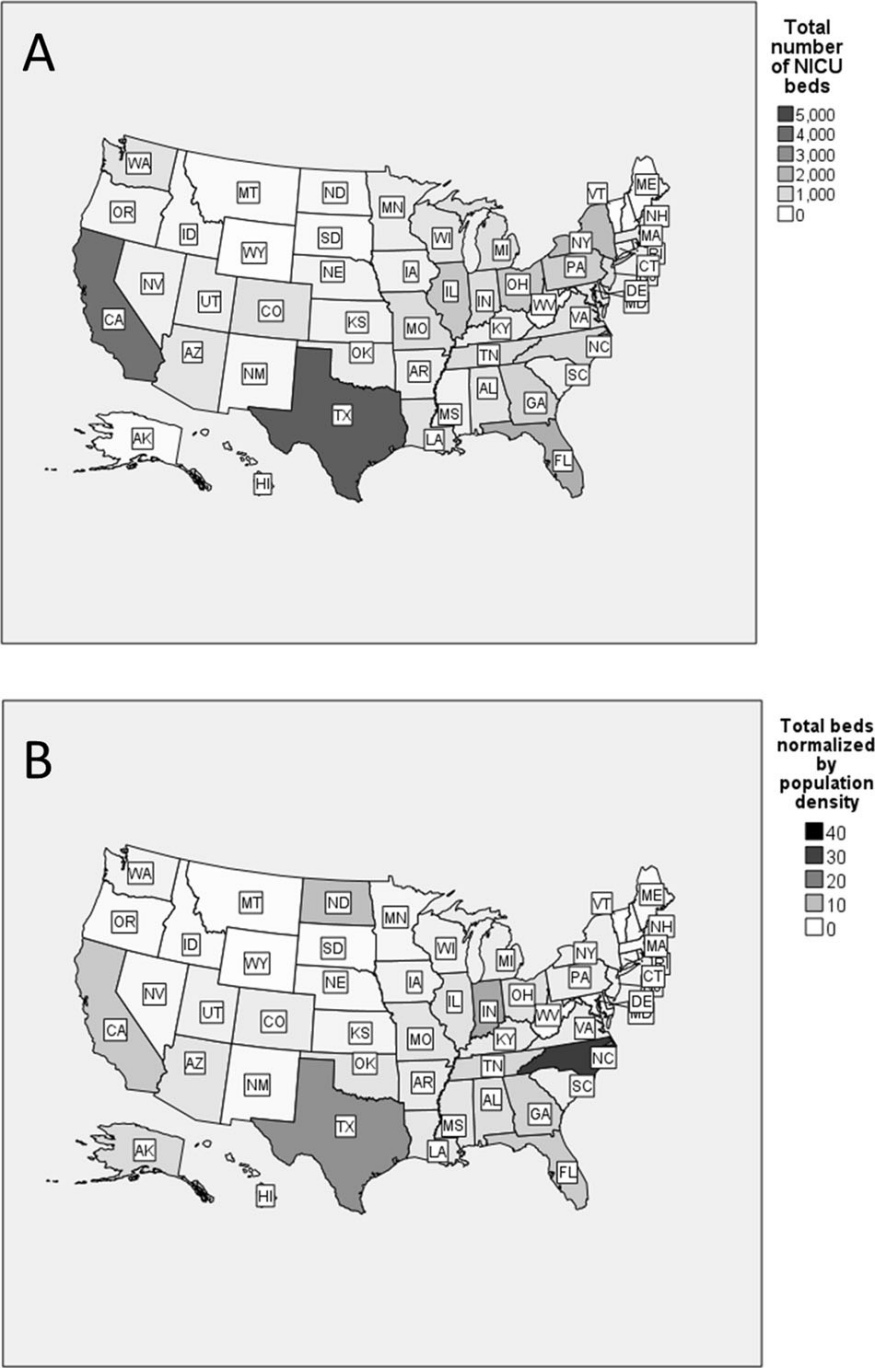
Distribution of NICU beds across different states in the US. Panel **A** includes the raw number of NICU beds across states. Panel **B** is the distribution of NICU beds normalized to population density (beds per number of people by 0.1 square miles). Legend entries denote colors within the shade spectrum for the cloropleth map, along with their corresponding lower threshold value.

See Fig. 2 for the distribution of NICU beds according to state and number of beds normalized to population density (beds per number of people by 0.1 square miles). While Texas and California had the largest number of NICU beds, North Carolina had the greatest number of NICU beds per capita.

Table 2 summarizes the relationships of NICU level with hospital and population characteristics using ordinal logistic regression; both unadjusted and multivariable-adjusted associations are displayed. The multivariable model includes independent variables that are statistically significantly associated with NICU level when adjusted for other variables in the model. On multivariable modeling, statistically significant positive associations with higher NICU level remained for higher number of NICU beds (*p* < 0.0001), being embedded in a free-standing children’s hospital or part of a children’s hospital (compared to a non-children’s hospital, *p* < 0.0001), being part of an academic medical center (*p* = 0.006), and higher population density (*p* < 0.0001). CON legislation was inversely associated with NICU level (*p* = 0.023); NICUs in states subject to CON legislation were more likely to be of lower NICU levels. There was also significant variation in NICU level by region (*p* = 0.01) with the Northeast and Southeast having more NICUs with high acuity level.

**Table 2:** Relationships between higher level of NICU and hospital and population factors (ordinal logistic regression).

NICU Factors	Level II *N* (%) Or Median (IQR)	Level III *N* (%) Or Median (IQR)	Level IV *N* (%) Or Median (IQR)	Unadjusted OR (95% CI); *p*-value	Multivariable adjusted OR (95% CI); *p*-value
Number of beds (OR reported per bed)	8 (6)	25 (21)	55 (30)	1.13 (1.11, 1.14) *p* < 0.0001	1.11 (1.10, 1.13) *p* < 0.0001
Free-standing children’s hospitals					
Yes, free-standing children’s hospital	1 (0.2%)	15 (2.1%)	52 (34.2%)	114.5 (60.0, 218.6)	42.6 (17.4, 104.1)
Not freestanding, but part of children’s hospital	3 (0.5%)	81 (11.5%)	66 (43.4%)	28.6 (18.4, 44.4)	8.3 (4.6, 14.9)
Not children’s hospital	566 (99.3%)	606 (86.3%)	34 (22.4%)	Referent *p* < 0.0001	Referent *p* < 0.0001
Academic and non-academic regional hospitals					
Academic	13 (2.3%)	79 (11.2%)	85 (55.9%)	Referent	Referent
Community	512 (91.1%)	554 (78.9%)	46 (30.3%)	0.06 (0.04, 0.09)	0.42 (0.25, 0.71)
Regional non-academic	23 (4.1%)	40 (5.7%)	19 (12.5%)	0.20 (0.12, 0.36)	0.40 (0.20, 0.81)
County/Indian health service/Military	14 (2.5%)	29 (4.1%)	2 (1.3%)	0.10 (0.05, 0.20) *p* < 0.0001	0.69 (0.27, 1.72) *p* = 0.006
Governmental and non-profit hospitals (vs. proprietary)					
Non-profit control	388 (69.2%)	468 (67.3%)	112 (77.8%)	Referent	
Proprietary control	110 (19.6%)	130 (18.7%)	13 (9.0%)	0.78 (0.60, 1.02)	
Governmental control	63 (11.2%)	97 (14.0%)	19 (13.2%)	1.15 (0.85, 1.56) *p* = 0.083	
**Population Factors**					
Geographic region					
Northeast	95 (16.7%))	133 (19.0%)	30 (19.7%)	Referent	Referent
Southeast	152 (26.7%)	176 (25.1%)	39 (25.7%)	0.84 (0.62, 1.14)	1.00 (0.66, 1.51)
Midwest	149 (26.1%)	139 (19.8%)	26 (17.1%)	0.65 (0.48, 0.90)	0.58 (0.37, 0.91)
Southwest	66 (11.6%)	82 (11.7%)	26 (17.1%)	1.04 (0.72, 1.51)	0.82 (0.48, 1.43)
West	108 (19.0%)	172 (24.5%)	31 (20.4%)	1.03 (0.75, 1.41)	1.12 (0.71 1.75) *p* = 0.011
Population density (people per square mile) (OR reported per 1000 pepole per square mile)	2295 (2138)	3261 (3014)	3630 (3607.5)	1.08 (1.06, 1.10) *p* < 0.0001	1.07 (1.04, 1.11) *p* < 0.0001
Percent of population living in poverty (OR reported per 10% living in poverty)	13.9 (11.8)	16.6 (9.9)	18.0 (7.4)	1.48 (1.29, 1.69) *p* < 0.0001	
Percent of population under 5 years old (OR reported per 10% under 5 years old)	6.5 (1.6)	6.4 (1.3)	6.4 (1.0)	0.71 (0.33, 1.54) *p* = 0.39	
Percentage of population from racial/ethnic minorities (OR reported per 10% minority)	34.2 (36.4)	46.9 (34.9)	54.5 (32.0)	1.20 (1.15, 1.26) *p* < 0.0001	
Subject to CON (certificate of need) legislation					
Yes	306 (53.7%)	384 (54.7%)	77 (50.7%)	1.02 (0.84, 1.25)	0.69 (0.50, 0.95)
No	264 (46.3%)	318 (45.3%)	75 (49.3%)	Referent *p* = 0.84	Referent *p* = 0.023

Data analyzed by ordinal logistic regression with NICU level as the dependent variable. Independent variables are reported by NICU level as median (IQR) for continuous variables and frequency (percent) for categorical variables. Associations reported as odds ratios (OR) with 95% confidence interval (CI). The multivariable ordinal logistic regression model includes independent variables that are each significantly associated (p < 0.05) when adjusted for other variables in the model. Variables that are not significant on univariate or multivariate modeling are not included.

**Fig. 3:**
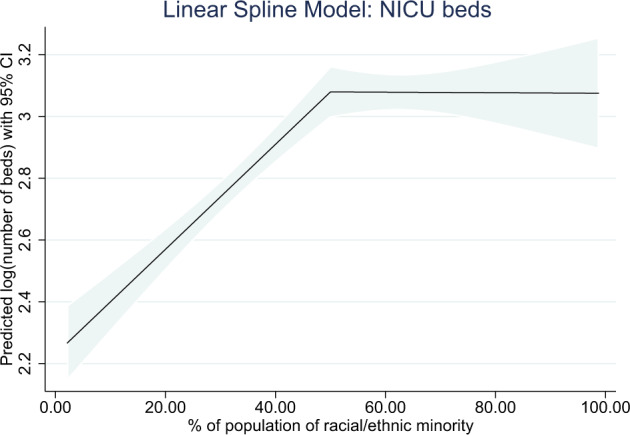
Linear spline demonstrating steady increase in number of NICU beds and then plateau when the community is > 50% minorities.

Table 3 summarizes the unadjusted and multivariable-adjusted relationships between number of NICU beds (log transformed) and hospital and population characteristics. The multivariable model includes independent variables that are statistically significantly associated with NICU level when adjusted for other variables in the model. On multivariable modeling, higher number of NICU beds was positively associated with higher NICU level (*p* < 0.0001), being in a free-standing children’s hospital or part of a children’s hospital (*p* < 0.0001), being in an academic hospital (*p* = 0.0001), and being in a state subject to CON legislation (*p* = 0.046). Using a linear spline, the percent of the population comprised of racial/ethnic minorities showed a non-linear association with number of beds; the number of beds was positively associated with higher percentages of minorities up to 50% minorities, but was not associated when the population comprised 50% and higher minorities (see Fig. 3). The number of beds also significantly differed by region (*p* = 0.019), with hospitals with higher numbers of NICU beds in the Northeast.

**Table 3:** Relationships between log(number of NICU beds) and hospital and population factors.

NICU Factors	Median (IQR) number of beds	Unadjusted regression coefficient (95% CI), *p*-value, R^2^	Multivariable adjusted regression coefficient (95% CI)	Adjusted *p*-value
NICU level				
II	8 (6)	1.009 (0.963, 1.056)	0.869 (0.813, 0.925)	<0.0001
III	25 (21)	*p* < 0.0001		
IV	55 (30)	R^2^ = 0.560		
Free-standing children’s hospitals				
Yes, free-standing children’s hospital	56 (40)	1.284 (1.097, 1.471)	0.064 (−0.095, 0.222)	<0.0001
Not freestanding, but part of children’s hospital	45 (24)	1.135 (1.005, 1.265)	0.279 (0.169, 0.389)	
Not children’s hospital	15 (18)	Referent *p* < 0.0001 R^2^ = 0.237	Referent	
Academic and non-academic regional hospitals				
Academic	46 (30)	Referent	Referent	0.0001
Community	15 (18)	−1.032 (−1.158, −0.905)	−0.187 (−0.293, −0.082)	
Regional non-academic	33.5 (48)	−0.487 (−0.696, −0.279)	−0.021 (−0.171, 0.130)	
County/Indian health service/Military	20 (15)	−1.029 (−1.290, −0.768) *p* < 0.0001 R^2^ = 0.164	−0.372 (−0.562, −0.182)	
Governmental and non-profit hospitals (vs. proprietary)				
Non-profit control	18 (25)	Referent		
Proprietary control	15 (18)	−0.142 (−0.262, −0.021)		
Governmental control	21 (28)	0.093 (−0.046, 0.232) *p* = 0.015 R^2^ = 0.006		
**Population Factors**				
Geographic region				
Northeast	16 (23)	Referent	Referent	0.019
Southeast	16 (25)	0.005 (−0.134, 0.144)	0.056 (−0.035, 0.147)	
Midwest	16.5 (27)	−0.089 (−0.233, 0.055)	0.145 (0.044, 0.246)	
Southwest	21 (28)	0.170 (0.001, 0.338)	0.192 (0.067, 0.317)	
West	19 (19)	0.023 (−0.121, 0.167) *p* = 0.042 R^2^ = 0.007	0.108 (0.004, 0.212)	
Population density (per 1000 people/square mile)				
		0.030 (0.020, 0.039) *p* < 0.0001 R^2^ = 0.026		
Percent of population living in poverty (per percentage unit)				
Linear spline - Regression coefficient for:	14 (18)	0.055 (0041, 0.069)		
% in poverty <15%	22 (28)	0.005 (−0.007, 0.017)		
15%≤% in poverty <30% % in poverty ≥30%	22 (27)	−0.054 (−0.094, −0.014) *p* < 0.0001 R^2^ = 0.064		
Percent of population under 5 years old (per percentage unit)				
Linear spline – Regression coefficient for:				
% under 5 < 7%	18 (24)	0.056 (0.006, 0.105)		
7%≤% under 5 < 10%	16 (25)	−0.185 (−0.289, −0.080)		
% under 5 ≥ 10%	14 (13)	0.220 (−0.190, 0.631) *p* = 0.0045 R^2^ = 0.009		
Percent of population of ethnic and racial minorities (per percentage unit)				
Linear spline – Regression coefficient for:				
% minority <50%	15 (21)	0.017 (0.013, 0.021)	0.007 (0.005, 0.010)	
% minority ≥50%	22 (28)	−0.000 (−0.005, 0.005) *p* < 0.0001 R^2^ = 0.077	−0.000 (−0.003, 0.003)	<0.0001
Subject to CON (certificate of need) legislation				
Yes	18 (25)	0.057 (−0.035, 0.148)	0.077 (0.001, 0.152)	0.046
No	18 (24)	Referent *p* = 0.22 R^2^ = 0.001	Referent	

Linear regression with log (number of beds) as dependent variable.

Multivariable model R^2^ for model with NICU level alone = 0.560; R^2^ for total multivariable model = 0.594.

The multivariable model includes independent variables that are statistically significantly associated with log(number of NICU beds) when adjusted for other variables in the model. Variables that are not significant on univariate or multivariate modeling are not included.

### City-specific relationships to population factors

After compiling data related to each city, we identified that each city with a NICU had between 1–18 NICUs, with 1–759 beds. See Supplemental Table for relationships of city-level log transformed number of beds related to population factors. On multivariable modeling on log-transformed number of beds, a linear spline (which fits a separate regression slope based on different levels of the independent variable) showed higher population density was related to more NICU beds (up to 20,000/square mile, *p* < 0.0001). A non-linear association with percentage living in poverty showed a positive association with number of beds (for <30% living in poverty) and an inverse association with number of beds (for 30% and more living in poverty) (*p* < 0.0001). A non-linear association with percent of population under 5 years showed an inverse association with number of beds (for <10% under 5 years old) and a positive association with number of beds for populations with 10% and more under 5 years old) (*p* = 0.004). The multivariable city-level model showed a non-linear association with percentage of the population comprised of racial/ethnic minorities; number of beds was positively associated with percentage minorities (for <70% minority populations) and inversely associated with percentage minorities (for populations with 70% and greater minorities) (*p* < 0.0001). There was significant variation in number of beds over geographic regions (*p* = 0.001). Being in a state subject to CON legislation was not related to the number of NICU beds (*p* = 0.35).

## Discussion

In this inaugural effort, we determined there were 1424 NICUs in the US that vary in level, size, and characteristics. Higher level and larger NICUs are more prevalent in academic medical centers in addition to being more likely to be housed within a children’s hospital. There are higher-level NICUs and NICUs with a larger number of beds in areas that have high population density, have a higher proportion of individuals identifying as a racial or ethnic minority, and a higher proportion of individuals experiencing poverty. After multivariable analysis, number of NICU beds was related to percentage of the population that consists of minorities, type of hospital (academic), geographic region (Northwest), whether part of a children’s hospital, and NICU level. After multivariable analysis, level of NICU remained associated with number of NICU beds, whether part of a children’s hospital, type of hospital (academic), geographic region (Northeast or Southeast) and population density. Although not significant on univariate analysis, CON legislation was discovered to be related to both NICU level and number of beds when controlling for other variables.

Here we highlight the significant variation in NICU levels, size, and other physical characteristics. While NICU level will define whether a NICU has the ability to provide a full range of respiratory support, NICUs at the same acuity level can also vary in terms of services offered and available specialties; such as use of extracorporeal membrane oxygenation, care for infants with diaphragmatic hernias, cardiac care, surgical intervention, and availability of associated follow-up clinics. Policies and procedures can vary across NICUs, including nurse-to-patient ratio, family presence policies, whether there are physical therapists, occupational therapists, and speech-language pathologists on staff in the NICU, and whether they are constructed of single-patient rooms or open bays. Further, culture within each NICU differs, including whether the NICU uses developmental care strategies, provides specialized parental education regarding caring for babies in the NICU, and whether evidence-based feeding practices such as cue-based feeding are integrated. The Vermont Oxford Network (VON), California Perinatal Quality Care Collaborative (CPQCC) and other statewide perinatal quality collaboratives, and The Children’s Hospitals Neonatal Consortium (CHNC) have moved the needle on aligning with collecting specific outcome measures across NICUs and have become central in working to improve the quality of NICU care. However, with a large number of NICUs and the significant variability among NICUs, parents are not able to identify these differences and often are faced with receiving care in the hospital that is part of their network, close to home, or known to them. By defining metrics of US-based NICUs, we can start to benchmark to improve access to care for all patients.

Preterm birth disproportionately affects women and families of color [34]. Further, infants with public insurance or living in rural areas are more likely to be born preterm [35]. With higher rates of preterm birth necessitating NICU care, the need for more NICUs with specialized higher levels of care would seem indicated. However, studies have demonstrated poor resource allocation and poor access to necessary medical care among these populations, leading to health inequities [36]. Unequal care within the NICU setting has been identified, and the downstream effects of systemic racism can have significant social impacts [36]. This study found there are more hospitals with higher numbers of NICU beds in areas that have higher percentages of individuals from racial and ethnic minorities and who are living in poverty. However, the number of NICU beds increases linearly with increased number of minorities until 50% minorities and then plateaus without any further increase in beds with increases in minorities in the community beyond 50%. This could potentially signal no additional increase in beds to account for the potential increase in utilization within communities with high density of minorities (50–100%). This study does not provide information regarding the quality-of-care metrics at each hospital. Other studies have focused on the differences in quality of care based on geographic locations and have found that despite the presence of healthcare resources, there are still disparities in the quality of care provided [36–39]. Simply having proximity to a higher level NICU was not necessarily an indicator of accessing those services, as healthcare choices are affected by other considerations such as insurance type [40]. Admissions to the NICU, where specialized care can be received, are higher when resources, such as insurance, exist [11]. Neonatal mortality is higher among certain groups of infants, such as the uninsured [41].

There appear to be geographical influences on types of NICUs present, and CON legislation was observed to relate to the types (both in terms of acuity level as well as number of beds) of NICUs in US locations. High-risk infants tend to have a longer length of stay as compared with other patient populations, so the presence of a NICU can potentially be profitable to a hospital. Having an extra layer of CON that ensures there is a need for additional NICU beds within a region before new NICUs can be opened would seem to discourage smaller units. This is consistent with our findings in which we identified relationships between CON legislation and type of NICU, with states who have CON legislation having a larger number of hospitals with higher acuity level and NICU beds. Interestingly, we did find that NICUs in the Northeast have NICUs with higher numbers of beds. NICUs in the Northeast and Southeast were more likely to have higher acuity level. Whether this reflects concentrated urban medical centers dedicated to regional care across large areas of land is unknown.

Another important concept underlying the designation of NICUs at different levels of care is regionalization. Regionalization is one strategy to reduce maternal and neonatal morbidity and mortality by facilitating early identification of high-risk pregnancies and establishing systems of care optimized for resource allocation [42]. Improvements in neonatal care were observed when the March of Dimes first designated different levels of NICU care and recommended that mothers be referred to the appropriate level of NICU based on their risk factors and the hospital’s commensurate resources and personnel [12, 42]. The benefits of regionalized care systems highlight improved competency of providers with consistent, high-volume exposure to high-risk neonates, as well as reductions in neonatal morbidity and mortality [43–45]. Despite consistent evidence of the benefits of regionalization, in the past few decades there has been increases in the number of smaller-volume NICUs as patient volumes and number of practicing neonatologists increases, maternal and neonatal medical technology advances and becomes more readily available, and hospitals strive to offer comprehensive services [44, 46]. There is some evidence that the growth in the number of NICUs in the last 30 years has contributed to de-regionalization of NICU care in many parts of the US [44, 46], with many Level II NICUs potentially accepting and caring for infants at high-risk with needs beyond the scope of care for that NICU [12]. A better understanding of how this has impacted neonatal mortality and morbidity is warranted. Having higher acuity NICUs with larger numbers of NICU beds in more locations could be a result of more regionalization in care, but this also requires further investigation.

This study had several limitations. It should be noted that univariate analyses were used without controlling for how the different variables reported influence each other. In addition, no adjustments to significance levels were made to account for multiple comparisons.

The nature of healthcare facilities, specifically NICUs, proved a rapidly changing target, with NICUs closing or expanding during the timeframe of this study. Data including level of NICU and number of beds were at risk of inconsistencies due to the ever-changing nature of the hospital care systems where NICUs often change number of beds (usually increasing capacity, but sometimes decreasing due to areas of the hospital closing). We suspect these changing numbers may be especially volatile during and following the COVID-19 pandemic, in which many units reallocated staffing and closed NICUs to provide space for other patients to accommodate hospital-wide demands [47]. Although this may represent a temporary impact on NICU beds, it is unclear how the pandemic may have shaped bed allocations temporarily or permanently. Inconsistencies in the multiple sources of information also existed. Phone calls directly to hospital systems proved helpful, but discrepancies in data from these reports also were observed and reconciled as needed. Survey and phone responses are also subject to bias and error, and there is a high likelihood of error due to reconciling multiple sources of information, including the use of websites that could be outdated.

The data available did not allow further differentiation of NICUs located in free-standing children’s hospitals associated with separate but proximately located adult hospitals as compared to those not associated with adult hospitals. These different ways that NICUs can exist within hospital systems is another important area for future inquiry. Additionally, there is a lot of noise in the way that beds can be reported. This study collected information on the number of approved NICU beds to define NICU size/volume, which does not account for NICU census. The number of approved beds were also put in context of the highest level of acuity at that hospital, with no differentiation of number of beds at each level of acuity within each hospital. In addition, our data is complicated by states having different classifications of NICU levels, and those with different requirements for each level [8]. Although the AAP created a new classification system to reduce variability across the United States [14], several states may not have adopted the new classifications at the time of data collection. In addition, a change in classification may have occurred during data collection, leading to inconsistencies in the labeling of NICU acuity level. Further, while we investigated the hospital characteristics in context of CON legislation in each state [48], we did not investigate findings in context of Centers of Excellence or other markers of quality, which is an important area for future inquiry.

The population statistics were derived from the city or county where the hospital was located; however, if a NICU resided near a geographic boundary between states, counties, or cities, the correlations calculated may not properly account for true patient population in a particular NICU. Further, the population surrounding the hospital (in its city or county) may not relate to the geographic markets for newborn or NICU care within the geographic area surrounding the hospital, meaning the data may not necessarily represent where the NICU population was drawn from. Because we investigated each NICU as a unit, specific geographical areas with a large density of NICUs may confound the results, as the characteristics of one unit in the area may influence the others. Further, this study did not account for regionalization, which could have impacted the number, size, and type of NICUs in a given area. Although we were able to demonstrate relationships between higher population of minorities and people living in poverty associated with a greater number of NICU beds, this does not imply greater access to NICU services, and warrants further investigation.

## Conclusion

Despite the limitations of this study, this is the first report we are aware of that aims to define characteristics of US-based NICUs. It can be used for benchmarking and understanding variation in NICU care across the US. Furthermore, it is our hope and intention that it sparks renewed focus on NICU care and is used to better define metrics of quality and safety to improve the care that all infants and families receive. Data developed as part of this manuscript are available for review and update to enable easier analysis in the future at nicudata.com.

## Supplementary information

Relationships between city-level log(number of NICU beds) and population factors.

## Data Availability

A partial data set supporting the findings of this study will be available at https://www.nicudata.com. The full data set can be requested by contacting the corresponding author at any time.

## References

[1] Kim Y, Ganduglia-Cazaban C, Chan W, Lee M, Goodman DC (2021). Trends in neonatal intensive care unit admissions by race/ethnicity in the United States, 2008-2018. Sci Rep.

[2] Martin JA, Hamilton BE, Osterman MJ. Births in the United States, 2021. NCHS Data Brief. 2022;442:1-8. 36043891

[3] Schulman J, Braun D, Lee HC, Profit J, Duenas G, Bennett MV (2018). Association Between Neonatal Intensive Care Unit Admission Rates and Illness Acuity. JAMA Pediatr.

[4] Andrade SE, Scott PE, Davis RL, Li DK, Getahun D, Cheetham TC (2013). Validity of health plan and birth certificate data for pregnancy research. Pharmacoepidemiol Drug Saf.

[5] Northam S, Knapp TR (2006). The reliability and validity of birth certificates. J Obstet Gynecol Neonatal Nurs.

[6] Neonatal Intensive Care Market Size, Share, & Industry Analysis by Product (Neonatal Incubators, Neonatal Phototherapy Equipment, Neonatal Ventilators, Neonatal Monitors, and Others), by End User (Hospitals, and Specialty Clinics), and Regional Forecast, 2020-2027: Fortune Business Insights; 2020.

[7] Freedman S (2016). Capacity and Utilization in Health Care: The Effect of Empty Beds on Neonatal Intensive Care Admission. Am Econ J Econ Policy.

[8] Blackmon LR, Barfield WD, Stark AR (2009). Hospital neonatal services in the United States: variation in definitions, criteria, and regulatory status, 2008. J Perinatol.

[9] Lorch SA, Maheshwari P, Even-Shoshan O (2012). The impact of certificate of need programs on neonatal intensive care units. J Perinatol.

[10] CON - Certificate of Need State Laws: National Conference of State Legislatures; 2019. Available from: https://www.ncsl.org/research/health/con-certificate-of-need-state-laws.aspx.

[11] Harrison WN, Wasserman JR, Goodman DC (2018). Regional Variation in Neonatal Intensive Care Admissions and the Relationship to Bed Supply. The. J Ped.

[12] Newborn CoFa, Barfield WD, Papile L-A, Baley JE, Benitz W, Cummings J, et al. Levels Neonatal Care Ped. Pediatrics. 2012;130:587–97. 10.1542/peds.2012-1999 22926177

[13] Kroelinger CD, Okoroh EM, Goodman DA, Lasswell SM, Barfield WD (2018). Comparison of state risk-appropriate neonatal care policies with the 2012 AAP policy statement. J Perinatol.

[14] AAP NICU Verification Program: American Academy of Pediatrics; 2021. Available from: https://www.aap.org/en/patient-care/neonatal-care/.

[15] Newborn AAoPCoFA. (2012). Levels of neonatal care. Pediatrics..

[16] Apfeld JC, Kastenberg ZJ, Sylvester KG, Lee HC (2017). The effect of level of care on gastroschisis outcomes. The J Ped.

[17] Nair N, Patel RM, editors. The center-effect on outcomes for infants born at less than 25 weeks. Seminars in Perinatology; 2022: Elsevier. 10.1016/j.semperi.2021.151538 PMC9730551 34911651

[18] Phibbs CS, Bronstein JM, Buxton E, Phibbs RH (1996). The effects of patient volume and level of care at the hospital of birth on neonatal mortality. Jama..

[19] Phibbs CS, Baker LC, Caughey AB, Danielsen B, Schmitt SK, Phibbs RH (2007). Level and volume of neonatal intensive care and mortality in very-low-birth-weight infants. New England J Med.

[20] Lapcharoensap W, Gage SC, Kan P, Profit J, Shaw GM, Gould JB (2015). Hospital variation and risk factors for bronchopulmonary dysplasia in a population-based cohort. JAMA Pediatrics.

[21] Mohamed SA, Thota C, Browne PC, Diamond MP, Al-Hendy A. Why is Preterm Birth Stubbornly Higher in African-Americans? Obstet Gynecol Int J. 2014;1:78–79. 10.15406/ogij.2014.01.00019 PMC4402979 25905109

[22] Donoghue D, Lincoln D, Morgan G, Beard J (2013). Influences on the degree of preterm birth in New South Wales. Aust N Z J Public Health.

[23] Nguyen CA, Chernew ME, Ostrer I, Beaulieu ND. Comparison of healthcare delivery systems in low- and high-income communities. The American Journal of Accountable Care. 2019;7:11–18.

[24] Pineda R, DeGaetano S, Kindra M, Hand T, Craig J, Fernandez-Fernandez A (2019). Neonatal therapy: A survey of current practice. J Pediatr Rehabil Med.

[25] Pineda R, Lisle J, Ferrara L, Knudsen K, Kumar R, Fernandez-Fernandez A. Neonatal Therapy Staffing in the United States and Relationships to Neonatal Intensive Care Unit Type and Location, Level of Acuity, and Population Factors. Am J Perinatol. 2021. Epub ahead of print. 10.1055/a-1678-0002 34695863

[26] NICU Search: American Academy of Pediatrics; 2021. Available from: https://www.aap.org/en-us/advocacy-and-policy/aap-health-initiatives/nicuverification/Pages/NICUSearch.aspx.

[27] NICU Directory. Available from: https://neonatologysolutions.com/nicu-directory/.

[28] VON Member Map and Member List: Vermont Oxford Network; Available from: https://public.vtoxford.org/member-map/.

[29] The CHND Data Set: The Children’s Hospitals Neonatal Consortium; Available from: https://thechnc.org/home-page/leadership/the-chnd-data-set/.

[30] Fast Facts on US Hospitals, 2021: American Hospital Association; 2021. Available from: https://www.aha.org/statistics/fast-facts-us-hospitals.

[31] National Network of Perinatal Quality Collaboratives: National Institute for Children’s Health Quality; 2021. Available from: https://www.nichq.org/project/national-network-perinatal-quality-collaboratives.

[32] 2021 World Population by Country: World Population Review; 2021. Available from: https://worldpopulationreview.com.

[33] 2015-2019 ACS 5-year data profile: United States Census Bureau; Available from: https://www.census.gov/acs/www/data/data-tables-and-tools/data-profiles/.

[34] Culhane JF, Goldenberg RL (2011). Racial disparities in preterm birth. Semin Perinatol.

[35] 2020 March of Dimes Report Card: March of Dimes; 2020. Available from: https://www.marchofdimes.org/materials/US_REPORTCARD_FINAL_2020.pdf.

[36] Ravi D, Iacob A, Profit J (2021). Unequal care: Racial/ethnic disparities in neonatal intensive care delivery. Semin Perinatol.

[37] Hollenbach SJ, Thornburg LL, Glantz JC, Hill E (2021). Associations between historically redlined districts and racial disparities in current obstetric outcomes. JAMA Network Open.

[38] Edwards EM, Greenberg LT, Profit J, Draper D, Helkey D, Horbar JD. Quality of care in US NICUs by race and ethnicity. Pediatrics. 2021;148. 10.1542/peds.2020-037622 PMC8344358 34301773

[39] Lorch SA, Rogowski J, Profit J, Phibbs CS, editors. Access to risk-appropriate hospital care and disparities in neonatal outcomes in racial/ethnic groups and rural–urban populations. Seminars in Perinatology; 2021: Elsevier. 10.1016/j.semperi.2021.151409 PMC8184635 33931237

[40] Hebert PL, Chassin MR, Howell EA. The contribution of geography to black/white differences in the use of low neonatal mortality hospitals in New York City. Medical care. 2011;49:200–6. 10.1097/MLR.0b013e3182019144 21239954

[41] Morriss FH (2013). Increased risk of death among uninsured neonates. Health Serv Res.

[42] Ryan GM (1975). Toward improving the outcome of pregnancy Recommendations for the regional development of perinatal health services. Obstet Gynecol.

[43] Bronstein JM, Ounpraseuth S, Lowery CL (2020). Improving perinatal regionalization: 10 years of experience with an Arkansas initiative. J Perinatol.

[44] Handley SC, Lorch SA (2022). Regionalization of neonatal care: benefits, barriers, and beyond. J Perinatol.

[45] Walther F, Kuster DB, Bieber A, Rudiger M, Malzahn J, Schmitt J (2020). Impact of regionalisation and case-volume on neonatal and perinatal mortality: an umbrella review. BMJ Open.

[46] Bizzarro MJ, Gallagher PG (2020). Why so little progress in regionalization of perinatal care when transport of high-risk neonates remains a substantial risk?. J Perinatol.

[47] Chamberlain D. COVID-Related Nurse Shortage Leads to Temporary NICU Closure: A News Cafe; 2021. Available from: https://anewscafe.com/2021/08/03/redding/covid-related-nurse-shortage-leads-to-temporary-nicu-closure/.

[48] Elrod JK, Fortenberry JL (2017). Centers of excellence in healthcare institutions: what they are and how to assemble them. BMC Health Services research.

