# Supplementary material for: NICUs in the US: levels of acuity, number of beds, and relationships to population factors
Source: J Perinatol. 2023 May 19;43(6):796–805. doi: 10.1038/s41372-023-01693-6 (PMC10197033; doi:10.1038/s41372-023-01693-6)
Supplement: Supplementary file 1 — Relationships between city-level log(number of NICU beds) and population factors. [file 41372_2023_1693_MOESM1_ESM.docx]

Supplemental Table 1. Relationships between city-level log(number of NICU beds) and population factors.

| **Population Factors** | Median (IQR) number of beds | Unadjusted regression coefficient (95% CI), p-value, R^2^ | Multivariable adjusted regression coefficient (95% CI) | Adjusted p-value |
| --- | --- | --- | --- | --- |
| Geographic region (Southwest)  Northeast  Southeast  Midwest  Southwest  West | 16 (29.5)  16 (27)  16 (27)  24 (40)  20 (25) | Referent  0.028 (-0.178, 0.235)  -0.080 (-0.299, 0.139)  0.331 (0.044, 0.618)  0.083 (-0.136, 0.303)  p = 0.068  R^2^ = 0.009 | Referent  0.187 (-0.025, 0.398)  0.303 (0.089, 0.518)  0.576 (0.291, 0.861)  0.240 (0.034, 0.445) | 0.001 |
| Population density (per 1000*number/square mile)  Linear spline – Regression coefficient for:  Population density <20000/square mile  Population density ≥20000/square mile | 18 (30)  34 (187) | 0.099 (0.074, 0.125)  -0.042 (-0.101, 0016)  p<0.0001  R^2^ = 0.062 | 0.094 (0.064, 0.123)  -0.048 (-0.104, 0.008) | <0.0001 |
| Percent of population living in poverty (per percentage unit)  Linear spline - Regression coefficient for:  % in poverty <30%  % in poverty ≥30% | 18 (27)  30 (45) | 0.040 (0,030, 0.049)  -0.103 (-0.154, -0.051)  p<0.0001  R^2^ = 0.070 | 0.033 (0.024, 0.043)  -0.085 (-0.135, -0.036) | <0.0001 |
| Percent of population under 5 years old (per percentage unit)  Linear spline – Regression coefficient for:  % under 5 <7%  7% ≤ % under 5 <10%  % under 5 ≥10% | 19 (30)  16 (31)  14 (13) | 0.082 (0.013, 0.152)  -0.220 (-0.366, -0.075)  0.201 (-0.322, 0.724)  p = 0.013  R^2^ = 0.011 | -0.026 (-0.094, 0.041)  -0.223 (-0.365, -0.082)  0.426 (-0.057, 0.909) | 0.004 |

| Percent of population of ethnic and racial minorities (per percentage unit)  Linear spline – Regression coefficient for:  % minority <70%  % minority ≥70% | 17 (28)  28 (36) | 0.018 (0.014, 0.021)  -0.018 (-0.034, -0.002)  p<0.0001  R^2^ = 0.092 | 0.011 (0.007, 0.016)  -0.022 (-0.038, -0.006) | <0.0001 |
| --- | --- | --- | --- | --- |
| Subject to CON (certificate of need) legislation  Yes  No | 17.5 (30)  18.5 (27) | 0.067 (-0.073, 0.208)  Referent  p = 0.35  R^2^ = 0.001 |  |  |

Linear regression with log (number of beds) as dependent variable.

R^2^ for total multivariable model = 0.185
